# DC-SIGN (*CD209*) gene promoter polymorphisms in a Brazilian population and their association with human T-cell lymphotropic virus type 1 infection

**DOI:** 10.1099/vir.0.008367-0

**Published:** 2009-04

**Authors:** Simone Kashima, Evandra Strazza Rodrigues, Rochele Azevedo, Erick da Cruz Castelli, Celso Teixeira Mendes-Junior, France Keiko Nascimento Yoshioka, Israel Tojal da Silva, Osvaldo Massaiti Takayanagui, Dimas Tadeu Covas

**Affiliations:** 1 Regional Blood Center of Ribeirão Preto, University of São Paulo, Brazil; 2 School of Pharmaceutical Sciences of Ribeirão Preto, University of São Paulo, Brazil; 3 Division of Clinical Immunology, Faculty of Medicine of Ribeirão Preto, University of São Paulo, Brazil; 4 Department of Chemistry, School of Philosophy, Sciences and Literature, University of São Paulo, Brazil; 5 Faculty of Medicine of Ribeirão Preto, University of São Paulo, Brazil

## Abstract

This study evaluated four polymorphisms located in the DC-SIGN (*CD209*) gene promoter region (positions −336, −332 −201 and −139) in DNA samples from four Brazilian ethnic groups (Caucasians, Afro-Brazilian, Asians and Amerindians) to establish the population distribution of these single-nucleotide polymorphisms (SNPs) and correlated DC-SIGN polymorphisms and infection in samples from human T-cell lymphotropic virus type 1 (HTLV-1)-infected individuals. To identify *CD209* SNPs, 452 bp of the *CD209* promoter region were sequenced and the genotype and allelic frequencies were evaluated. This is the first study to show genetic polymorphism in the *CD209* gene in distinct Brazilian ethnic groups with the distribution of allelic and genotypic frequency. The results showed that −336A and −139A SNPs were quite common in Asians and that the −201T allele was not observed in Caucasians, Asians or Amerindians. No significant differences were observed between individuals with HTLV-1 disease and asymptomatic patients. However, the −336A variant was more frequent in HTLV-1-infected patients [HTLV-1-associated myelopathy/tropical spastic paraparesis (HAM/TSP), 80 %; healthy asymptomatic HTLV-1 carriers, 90 %] than in the control group (70 %) [*P*=0.0197, odds ratio (OR)=2.511, 95 % confidence interval (CI)=1.218–5.179). In addition, the −139A allele was found to be associated with protection against HTLV-1 infection (*P*=0.0037, OR=0.3758, 95 % CI=0.1954–0.7229) when the HTLV-1-infected patients as a whole were compared with the healthy-control group. These observations suggest that the −139A allele may be associated with HTLV-1 infection, although no significant association was observed among asymptomatic and HAM/TSP patients. In conclusion, the variation observed in SNPs −336 and −139 indicates that this lectin may be of crucial importance in the susceptibility/transmission of HTLV-1 infections.

## INTRODUCTION

Dendritic cells (DCs) are professional antigen-processing cells that act against invading pathogens (Banchereau & Steinman, 1998), being important as initiators and modulators of the immune response. DCs are efficient stimulators of T and B cells. As invading pathogens appear, DCs are produced in bone marrow and migrate to the tissues, where they capture and process the antigens. DC-SIGN [DC-specific intercellular adhesion molecule 3 (ICAM-3)-grabbing non-integrin], encoded by a member of the *CD209* gene family, is a receptor on DCs that binds to ICAM-3, which is expressed on T cells to facilitate the initial interaction between DCs and T cells (Soilleux *et al.*, 2002). Recently, Ceccaldi *et al.* (2006) demonstrated that DC-SIGN is an important co-factor that facilitates the fusion between HTLV-1-infected cells and DCs through ICAM-dependent mechanisms. In this context, DC-SIGN has been shown to be an important receptor of HTLV-1, Ebola virus, cytomegalovirus, dengue virus and other agents of infectious disease (Alvarez *et al.*, 2002; Halary *et al.*, 2002; Tailleux *et al.*, 2003; Tassaneetrithep *et al.*, 2003). In addition, Sakuntabhai *et al.* (2005) demonstrated that polymorphisms in the *CD209* gene promoter region (position −336) affect its activity by modifying gene transcription significantly. Therefore, variations in this region may have an influence on the pathogenesis of human infectious diseases.

Human T-cell lymphotropic virus type 1 (HTLV-1) was the first retrovirus isolated from humans (Poiesz *et al.*, 1980). The virus has tropism for T cells and is related to clinical disorders such as adult T-cell leukaemia/lymphoma (Hinuma *et al.*, 1981; Yoshida *et al.*, 1982) and HTLV-1-associated myelopathy/tropical spastic paraparesis (HAM/TSP) (Gessain *et al.*, 1985; Osame *et al.*, 1986). Other inflammatory syndromes are also related to HTLV-1, such as polymyositis (Morgan *et al.*, 1989), uveitis (Mochizuki *et al.*, 1992) and infective dermatitis (LaGrenade *et al.*, 1990). Nevertheless, only 2–3 % of infected individuals develop these disorders. These distinct clinical disorders may depend on the type and magnitude of the host immune response to HTLV-1 antigens and the site/organ where the inflammatory reactions occurred. Several studies have demonstrated that genetic heterogeneity may determine the molecular basis of predisposition to HTLV-1 infection (Plancoulaine *et al.*, 2006; Pociot *et al.*, 1992; Turner *et al.*, 1997). Thus, is important to point out that host genetic factors may contribute to disease susceptibility, although the precise mechanisms involved are not completely understood.

On this basis, the objective of the present study was to investigate the distribution of four single-nucleotide polymorphisms (SNPs) located in the *CD209* gene promoter at positions −336, −332, −201 and −139 in four distinct Brazilian ethnic groups (Caucasians, Afro-Brazilians, Asians and Amerindians). We also compared SNP distribution between HTLV-1-infected individuals and a control group to determine whether the frequencies of *CD209* polymorphisms influence the susceptibility of HTLV-1-infected individuals to developing HAM/TSP.

## METHODS

### Study design

#### Brazilian ethnic groups.

Blood samples were collected from four ethnically distinct Brazilian populations: 45 from Caucasian individuals (46.7 % males; mean age, 38.7 years), 25 from Afro-Brazilians (60.0 % males; mean age, 34.4 years), 28 from Asians (57.1 % males; mean age, 40.7 years) and 25 from Amerindians (69.6 % males; it was not possible to determine the age of the Amerindians). The Caucasian, Afro-Brazilian and Asian groups were selected at random from blood donors of the Regional Blood Center of Ribeirão Preto and Núcleo de Haemoterapia of Franca, Brazil. Only individuals who reported the absence of racial admixture in their four grandparents were included in the study. The Amerindian samples were collected from Xavante Amerindians who live in Campinápolis city, Mato Grosso state, Brazil.

#### HTLV-1-seropositive individuals.

The HTLV-1-seropositive patients studied here were detected among blood-donor candidates of the Regional Blood Center of Ribeirão Preto and among patients of the Neurology Department of the University Hospital, São Paulo, Brazil. Diagnosis of HTLV-1/2 infection was established by antibody screening of serum/plasma samples using an enzyme immunoassay (rp21e-enhanced EIA; Cambridge Biotech), followed by confirmation by PCR (*tax* and LTR regions). The total HTLV-1 group consisted of 66 patients (62.1 % females; mean age, 47.5 years), most of whom were Caucasian (77.3 %). The HTLV-1 group was classified into healthy asymptomatic HTLV-1 carriers (HAC) and patients with HAM/TSP. The HAC group consisted of 33 individuals (54.5 % females; mean age, 40.5 years) and the HAM/TSP group consisted of 33 patients (69.7 % females; mean age, 53.2 years). Both groups were mainly of Caucasian origin (75.8 and 78.8 %, respectively). The admixed control group consisted of 32 randomly selected blood donors from the same geographical region as the HTLV-1-positive individuals described above, with a mean age of 34.8 years (84.4 % males); the ethnic background was evaluated based on skin colour and individuals were mainly Caucasians (62.5 %) and African descendants (34.4 %). The study was approved by the Institutional Ethics Committee of the University Hospital, Faculty of Medicine of Ribeirão Preto, University of São Paulo (process number 7639/2005) and all subjects gave written, informed consent to participate.

#### DNA extraction.

A 5 ml blood sample was collected into EDTA-containing tubes and plasma was separated under aseptic conditions by centrifugation at 900 ***g*** for 10 min at 4 °C. The white-cell layer was aspirated together with the red blood cells and placed in sterile 15 ml polypropylene tubes. DNA was extracted from the buffy coat by using a Super Quick Gene DNA Isolation kit (Analytical Genetic Testing Center, CO, USA) and diluted in 200 μl ultrapure water. All samples were quantified and diluted to 0.1 μg DNA μl^−1^.

#### Amplification reaction.

For genotyping, the *CD209* gene promoter was amplified in all samples under study by using the primers 5′-CAAAAATGAGGACAGCAGCA-3′ and 5′-CTCCAAGGAACCAAGACTGC-3′, which delimit the promoter region between nt −423 and 28 (defined from GenBank accession no. NC_000019) and amplify a fragment of 452 bp. Reactions were performed in 25 μl mixtures containing 100 ng DNA, 1.0 U *Taq* DNA polymerase (Invitrogen Life Technologies), 50 mM KCl, 20 mM Tris/HCl (pH 8.3), 1.5 mM MgCl_2_, 0.2 mM each dNTP and 0.3 pmol each primer. Thermocycling was performed in a GeneAmp PCR system 9700 (Applied Biosystems) under the following conditions: an initial cycle at 95 °C for 5 min and 35 cycles at 95 °C for 40 s, 62 °C for 40 s and 72 °C for 60 s, with a final extension step of 72 °C for 10 min. The amplified products were analysed by 1 % agarose gel electrophoresis followed by ethidium bromide staining.

#### DNA sequencing.

Sequencing reactions were performed using PCR primers and a DYEnamic ET dye terminator kit (GE Healthcare UK) according to the manufacturer's instructions. Electrophoresis was carried out using an automated apparatus MegaBace DNA sequencing system 1000 (Amersham Biosciences). The electropherograms were analysed by using Sequence Analyser software version 1 (Amersham Biosciences). Consensus sequences were obtained and compared by multiple alignments, using GenBank accession no. NC_000019 as the prototype sequence.

#### Sequences analysis.

Electropherograms were processed by using the base-calling program Phred (Ewing & Green, 1998; Ewing *et al.*, 1998), resulting in the generation of files including information about base content and base quality of each sequence. The sequences were aligned pairwise to the specific genomic region by using the Cross Math program (http://www.phrap.org). Multiple alignments were produced by using the polybayes multiple-alignment algorithms (Marth *et al.*, 1999). The SNPs were detected in the highly similar regions of multiple anchored alignments by using the PolyPhred program (Nickerson *et al.*, 1997). An automated pipeline was prepared to process the above steps, integrated with a number of in-house-developed Perl scripts.

#### Statistical analysis.

Ethnic differences between patients and controls were evaluated by an exact test that employed the Metropolis algorithm to obtain an unbiased estimate of the exact *P* value and its standard error by using R×C software (http://www.marksgeneticsoftware.net/rxc.htm). This same method was used to compare the frequencies of SNPs among the ethnic groups. Allelic frequencies and observed heterozygosity were computed by a direct counting method. Adherences of genotypic proportions to expectations under Hardy–Weinberg equilibrium were tested by the exact test of Guo & Thompson (1992), employing Arlequin software version 3.11 (Excoffier *et al.*, 2005). genepop version 3.4 software (Raymond & Rousset, 1995a) was used to perform a pairwise exact test of population differentiation (Raymond & Rousset, 1995b) based on allele and genotype frequencies. The Arlequin 3.11 software was also used to perform an exact test of population differentiation based on haplotype frequencies.

The presence of a significant association between loci was determined by means of a likelihood-ratio test of linkage disequilibrium (Excoffier & Slatkin, 1998) using the Arlequin program, version 3.1 (Excoffier *et al.*, 2005). Given the positive association but unknown gametic phase, the phase (Stephens *et al.*, 2001) and em (Excoffier & Slatkin, 1995) algorithms were also used to reconstruct *CD209* haplotypes.

The allele, genotype and haplotype frequencies were compared by using Fisher's exact test implemented in GraphPad InStat 3 software, which was also used to estimate the odds ratio (OR) and its 95 % confidence interval (CI).

## RESULTS

### *CD209* promoter polymorphisms in the Brazilian population

We investigated four polymorphisms located in the *CD209* promoter region at positions −336, −332, −201 and −139 that are known to be associated with susceptibility/resistance to pathogens. The allelic frequencies of these SNPs for Brazilian ethnic groups and other populations previously described are presented in Table 1. All loci evaluated in the *CD209* promoter region fitted the Hardy–Weinberg equilibrium expectations for all groups.

The −336G allele showed higher frequencies in Afro-Brazilians (42.0 %) and Caucasians (26.7 %) than in Asians (5.4 %). In addition, this allele was not observed in the Amerindian group, and the admixed control group presented frequencies similar to those of the Caucasian group (Table 1). SNP −332A was present at low frequencies in almost all ethnic groups from Brazil, except the Amerindians. Due to its low frequency among the groups, the control group did not present SNP −332A (Table 1). The −332A allele was identified at higher frequencies in the Asian group (10.7 %) compared with Afro-Brazilian (4.0 %) and Caucasian (4.4 %) individuals.

We also analysed the −201T/G position, which was absent in all ethnic groups from Brazil except for the Afro-Brazilian population (6.0 %) and was present in the control group at a frequency of 1.6 %. Analysis of the −139A/G position showed that the allelic frequency of SNP −139A was higher in Asians (62.5 %) than in Afro-Brazilians (26.0 %), Caucasians (35.6 %) and Amerindians (30.0 %). The Brazilian control group showed frequencies similar to those of the Caucasian group (Table 1).

An exact test of allelic frequency differentiation, performed based on the frequencies of SNPs −336 and −139, did not reveal any difference between Brazilian Caucasians and European Caucasians (Barreiro *et al.*, 2006a) (*P*=0.2835± 0.0060), between Afro-Brazilians and Zimbabweans (Boily-Larouche *et al.*, 2007) (*P*=0.5503±0.0080) or sub-Saharan Africans (Barreiro *et al.*, 2007) (*P*=0.5018±0.0071), and between Brazilian Asians and Asians (Barreiro *et al.*, 2006a) (*P*=1.0000±0.0000).

The presence of a significant association between SNPs was estimated by a likelihood-ratio test of linkage disequilibrium considering all of the samples from the control admixed and ethnic groups. A positive association was detected between SNP −336 and SNP −201 (*P*=0.0138±0.0005) and –139 (*P*=0.0000±0.0000), and between SNP −332 and SNP −139 (*P*=0.0028±0.0002). Given the positive association but unknown gametic phase, the phase and em algorithms were used to reconstruct *CD209* haplotypes (Table 2). The same haplotype was estimated in all samples by the phase and em algorithms, with mean probabilities of 0.9753 and 0.9837, respectively.

### HTLV-1 individuals

All HTLV-1 individuals were evaluated for clinical status. Of these, 50 % were HAC and 50 % had HAM/TSP. None of the patients showed positive serology for other retroviruses, e.g. human immunodeficiency virus type 1 (HIV-1) or HTLV-2. The sex, age and ethnic background distribution of the blood donors are shown in Table 3. The HAM/TSP group showed a homogeneous distribution between females and males. A high frequency of infection among Caucasian individuals was detected in both the HAC and HAM/TSP groups (Table 3).

Table 4 shows the genotype and allele frequencies for SNPs −336, −332, −139 and −201 among the HTLV-1-infected individuals (HAC and HAM/TSP) and the admixed-control group. It is important to note that the ethnic background of the HTLV-1 groups, including HAC and HAM/TSP, did not differ from that of the controls (*P*=0.1125±0.0079, *P*=0.2812±0.0089 and *P*=0.0916± 0.0062, respectively).

An exact test of population differentiation based on the allele and genotype frequencies revealed that the total HTLV-1 group showed differences in the allele (*P*=0.0035) and genotype (*P*=0.0055) frequencies compared with the controls. However, no differences were found between the HAC and HAM/TSP groups. In order to detect a possible influence of specific alleles or genotypes, 2×2 contingency tables were created to compare the frequencies using Fisher's exact test.

The −336A allele was found to be associated with susceptibility to HTLV-1 infection and the −336G allele was found to be associated with protection against infection when the HTLV-1-infected patients as a whole were compared with the healthy-control group (*P*=0.0197, OR=2.511, 95 % CI=1.218–5.179). Likewise, the −336GG genotype was found to be associated with protection against HTLV-1 infection when the HTLV-1 group as a whole was compared with the healthy-control group (*P*=0.0030, OR=0.03759, 95 % CI=0.002008–0.7038). On this basis, it is plausible to assume that the −336G allele is the variant conferring resistance to HTLV-1 infection. When the asymptomatic HTLV-1-infected patients were compared with the HAM/TSP group, no difference was found regarding this polymorphism (*P*=0.1353) (Table 4). However, the frequency of the −336G allele was significantly lower in the asymptomatic HTLV-1-infected group than in healthy controls (*P*=0.0036, OR=0.2368, 95 % CI=0.0874–0.6414) (Table 4). Likewise, the frequencies of both homozygous genotypes for the −336 locus (AA and GG) in the asymptomatic group were different from those of the healthy-control group, the first one showing a higher frequency (*P*=0.0332, OR=3.500, 95 % CI=1.134–10.806) and the second one a lower frequency (*P*=0.0244, OR=0.07436, 95 % CI=0.003948–1.411) in patients (Table 4). Genotype −336GG was also under-represented in the HAM/TSP group compared with the healthy-control group (*P*=0.0244, OR=0.07436, 95 % CI=0.003948–1.411) (Table 4). Nevertheless, it is reasonable to infer that these are probably hitchhiking effects of the protective influence of the −336G allele against HTLV-1 infection.

Regarding SNPs −332 and −201, no differences for the allele and genotype frequencies were found among the groups (Table 4). The −139A allele was found to be associated with protection against HTLV-1 infection (*P*=0.0037, OR=0.3758, 95 % CI=0.1954–0.7229) when the HTLV-1-infected patients as a whole were compared with the healthy-control group. Likewise, the −139AA genotype was associated with protection against HTLV-1 infection (*P*=0.0050, OR=0.1116, 95 % CI=0.02168–0.5745). No differences were found between the asymptomatic group and the HAM/TSP group regarding this polymorphism (*P*=0.6666). However, the frequency of the −139A allele was considerably lower in the asymptomatic and HAM/TSP groups compared with the healthy-control group (*P*=0.0067, OR=0.3248, 95 % CI=0.1459–0.7230 and *P*=0.0377, OR=0.4299, 95 % CI=0.2006–0.9209, respectively) (Table 4). Likewise, the frequency of the −139AA genotype was lower in the asymptomatic group compared with the control (*P*=0.0048, OR=0.05075, 95 % CI=0.002766–0.9310) (Table 4). However, these results may also be a hitchhiking effect of the protective influence of the −139A allele against HTLV-1 infection.

An exact linkage-disequilibrium test taking into account all samples revealed a high linkage disequilibrium between SNPs −336 and −139 (*P*=0.0000±0.0000), which may indicate a possible additive or co-dependent effect for both polymorphisms in the resistance/susceptibility to HTLV-1. In order to determine such interactions, haplotypes for each sample were estimated by two distinct methods without taking into account any prior information. Table 5 shows the frequency of each haplotype found in HTLV-1-infected patients and in the control group. An exact test of differentiation based on haplotype frequencies revealed a difference between the HTLV-1 group as a whole and the healthy-control group (*P*=0.0000±0.0000). As observed for the allele and genotype frequencies, there was no difference between the HAM/TSP and the asymptomatic group (*P*=0.3328±0.0206), but both HTLV-1 groups did differ from the healthy-control group (*P*=0.0005±0.0004 and *P*=0.0000±0.0000, respectively).

In fact, when the HTLV-1 group as a whole was compared with the healthy-control group, haplotypes −336G/−332G/−201G/−139G and −336A/−332G/−201G/−139A were found to be associated with protection against HTLV-1 infection (*P*=0.0045, OR=0.3276, 95 % CI=0.1524–0.7045 and *P*=0.0015, OR=0.3248, 95 % CI=0.1667–0.6327, respectively), whilst haplotype −336A/−332G/−201G/−139G was associated with susceptibility to HTLV-1 infection (*P*<0.0001, OR=4.145, 95 % CI=2.179–7.884). It is noteworthy that both haplotypes conferring protection carried a single dose of a protective allele (−336G or −139A), as well as a single dose of a susceptibility allele (−336A and −139G), whilst the susceptibility haplotype carried a double dose of the susceptibility alleles (−336A and −139G) (Table 5). In addition, following the same pattern of allele/genotype distribution among the groups, no difference was found between the HAM/TSP and asymptomatic groups (Table 5).

## DISCUSSION

We have described here for the first time the distribution of genetic polymorphisms in the *CD209* gene promoter (−336A/G, −332A/G, −201T/G and −139A/G positions) in distinct Brazilian ethnic groups and also their association with HTLV-1 infection.

In the Brazilian population studied here, the −336G allele was more frequent in the Afro-Brazilian group than in the Caucasian and Asian groups. However, the GG genotype was not detected in the Asian group and most subjects (86 %) were AA homozygotes, with only 13 % being heterozygotes. These data agree with previous studies that reported a higher frequency of the −336G allele in populations of African descent (Barreiro *et al.*, 2006a; Boily-Larouche *et al.*, 2007; Olesen *et al.*, 2007).

An interesting result was the identification of the −332A allele at high frequency in the Asian group (10.7 %). This variant has recently been described by Boily-Larouche *et al.* (2007) in a Zimbabwean population (2 %) as a probable position that can influence gene transcription.

The −201T allele was exclusively detected in the Afro-Brazilian population and has been reported only in African ethnic groups (Boily-Larouche *et al.*, 2007). This SNP was also observed here in the control group, demonstrating that, despite the miscegenation of the Brazilian population, the methodology used was efficient in separating the ethnic groups.

A high prevalence of the −139A allele was observed in the Asian population compared with the other ethnic groups. A similar result has been reported previously by Barreiro *et al.* (2006a) in a study assessing the frequency of this polymorphism in individuals of African, Asian and European descent. However, we have reported here for the first time the distribution of the −129G allele among Amerindians, which was surprisingly high (70.3 %).

As recently demonstrated, DCs play an important role in HTLV-1 infection, promoting contagion of T cells in both the *cis* and *trans* forms (Jones *et al.*, 2008). According to previous findings, HTLV-1-infected cells can use surface-adhesion molecules to regulate fusion with target cells, with the involvement of DC-SIGN and ICAM ligands. This mechanism could have consequences for the regulation of both DC infection and HTLV-1 dissemination, but also for immune regulation (Ceccaldi *et al.*, 2006). On this basis, in the present study we assessed SNP distribution in the promoter region of the *CD209* gene in an attempt to associate these polymorphisms with HTLV-1 infection. Infected individuals were divided into asymptomatic carriers and HAM/TSP carriers according to clinical condition, and the distribution of the polymorphisms was analysed in each group.

Regarding SNP −336, allele G was found to be less frequent among HAM/TSP (20 %) than HAC (9 %) individuals compared with controls (30 %). The absence of the GG genotype in the HTLV-1-infected group suggests that this −336G allele is a variant that confers protection against HTLV-1 infection. Thus, the presence of the −336G allele would result in lower DC susceptibility to HTLV-1 in the initial stages of infection, possibly protecting against HTLV-1 infectivity.

In agreement with the present results, the −336G allele has been associated with reduced DC-SIGN expression on the DC surface and in macrophages, as position −336 is located close to the major transcription site, possibly affecting binding of the transcription factor Sp1 and other factors that modulate transcriptional activity (Sakuntabhai *et al.*, 2005). On this basis, the antigen-presenting capacity of individuals who carry the −336G allele would be impaired, with a consequent alteration of the immune response. Regarding infectious diseases, position −336 in the promoter region of the *CD209* gene has been studied extensively. Vannberg *et al.* (2008) demonstrated that allele −336G is associated with protection against tuberculosis in the population of sub-Saharan Africa. Conversely, the −336G variant has been associated with susceptibility to HIV infection, haemorrhagic dengue and tuberculosis, suggesting that high levels of DC-SIGN expression permit better capture and processing of the antigens (Barreiro *et al.*, 2006a; Martin *et al.*, 2004; Sakuntabhai *et al.*, 2005).

Similarly, in the present study, polymorphisms at positions −332 and −201 of the promoter region of *CD209* were investigated and no significant association was found when the genotypes and alleles of infected individuals were compared with those of controls or when the HAC and HAM/TSP groups were compared.

For the SNP at position −139, allele A was found to be associated with protection against HTLV-1 infection when the infected and control groups were compared. Thus, these results suggest that this SNP may be associated with HTLV-1 transmission and not with development of the associated clinical manifestations. Previous studies have already demonstrated a higher frequency of allele −139A in individuals not infected with HIV compared with infected patients (Martin *et al.*, 2004). In another study, allele −139G was found to be associated with rapid progression of AIDS in a population of Japanese haemophiliacs. This SNP is located close to one of the binding sites of the transcription factor AP-1 in the promoter region of DC-SIGN and the substitution of one nucleotide close to this site can increase the level of expression of DC-SIGN, resulting in an accelerated progression of AIDS (Koizumi *et al.*, 2007).

Comparison of the HTLV-1 group and the healthy-control group showed that haplotypes −336G/−332G/−201G/−139G and −336A/−332G/−201G/−139A were associated with protection against HTLV-1 infection, whilst haplotype −336A/−332G/−201G/−139G was associated with susceptibility to HTLV-1 infection. These associations suggest that these polymorphisms may influence only the acquisition of HTLV-1 and not the associated diseases. Comparison of the HAM/TSP and asymptomatic groups with healthy controls revealed the presence of the same haplotypes conferring protection and susceptibility against HTLV-1, suggesting that these polymorphisms also have a hitchhiking effect on HTLV-1 acquisition.

In conclusion, the variations detected in the promoter region of the *CD209* gene may not be involved directly in triggering the development of HAM/TSP. However, the differences in SNP distribution observed between HTLV-1-infected individuals and controls suggest that these SNPs in the promoter region of *CD209* may be associated with the risk of HTLV-1 infection. Thus, the variations observed at positions −336 and −139 in the lectin DC-SIGN may be of crucial importance in the susceptibility/transmission of HTLV-1 infection.

## Figures and Tables

**Table 1. t1:** Allelic frequencies among Brazilian ethnic groups and previously reported frequencies of the *CD209* promoter polymorphisms

**Ethnic group**	n*	**Allelic frequency (%)**				**Reference**
		**−336G**	**−332A**	**−201T**	**−139G**	
**Brazilian groups**						
Caucasian	90	26.7	4.4	0.0	64.4	This study
Black	50	42.0	4.0	6.0	74.0	This study
Asian	56	5.4	10.7	0.0	37.5	This study
Amerindians	50	0.0	0.0	0.0	70.0	This study
Brazilian admixed control group	64	29.7	0.0	1.6	59.4	This study
**Other worldwide populations**						
Zimbabwean	200	45.0	2.0	11.5	74.0	Boily-Larouche *et al.* (2007)
Sub-Saharan African	82	37.8	–	–	87.8	Barreiro *et al.* (2006a)
South African (coloured)	1422	42.8	–	–	72.1	Barreiro *et al.* (2007)
Caucasian Canadian	200	18.0	–	0	69.5	Boily-Larouche *et al.* (2007)
Caucasian European	86	20.9	–	–	75.6	Barreiro *et al.* (2006a)
Thai	160	8.0	–	0	26.7	Sakuntabhai *et al.* (2005)
Asian	86	5.8	–	–	33.7	Barreiro *et al.* (2006a)
Granada, Spain	624	21.3	–	–	–	Núñez *et al.* (2006)
Medellin, Colombia	598	19.1	–	–	–	Gómez *et al.* (2006)
Pakistani	156	14.1	–	–	56.4	Barreiro *et al.* (2006b)
Guinea Bissau	680	49.9	–	–	76.0	Olesen *et al.* (2007)
Thailand	580	9.5	–	–	28.9	Wichukchinda *et al.* (2007)
Japanese†	216	3.7	–	–	20.4	Koizumi *et al.* (2007)

*n, Number of alleles. †HIV-1-infected Japanese haemophiliacs with the slow-progressor phenotype.

**Table 2. t2:** *CD209* haplotype frequencies in the Brazilian admixed population and among different ethnic groups

**Haplotype**	**Nucleotide at position**				**Haplotype frequency**				
	**−336**	**−332**	**−201**	**−139**	**Asians**	**Caucasians**	**African descendants**	**Admixed controls**	**Indians**
H1	A	G	G	G	0.3210	0.3889	0.3200	0.2969	0.7000
H2	G	G	G	G	0.0540	0.2556	0.3600	0.2813	0.0000
H3	A	G	G	A	0.5180	0.3000	0.2200	0.4063	0.3000
H4	G	G	T	G	0.0000	0.0000	0.0600	0.0156	0.0000
H5	A	A	G	A	0.1070	0.0444	0.0400	0.0000	0.0000
H6	G	G	G	A	0.0000	0.0111	0.0000	0.0000	0.0000

**Table 3. t3:** Baseline characteristics of the Brazilian ethnic population and individuals with HTLV-1

**Characteristic**	**Group**		
	**Control**	**HAC**	**HAM/TSP**
*n* (total=98)	32	33	33
Mean age (years) [range]	34.8 [22–57]	40.5 [19–64]	53.2 [31–70]
Sex [%]			
Male	27 [84.4]	15 [45.5]	10 [30.3]
Female	5 [15.6]	18 [54.5]	23 [69.7]
Ethnic background [%]			
Non-white	11 [34.4]	5 [15.2]	7 [21.2]
Caucasian	20 [62.5]	25 [75.8]	26 [78.8]
Asian	0 [0]	1 [3.0]	0 [0]
Not determined	1 [3.1]	2 [6.0]	0 [0]

**Table 4. t4:** Allele and genotype frequencies among HTLV-1-infected patients (HAC and HAM/TSP) and a healthy-control group

**Allele**	**Genotype**	**Controls (*n*=32)**	**HAM/TSP (*n*=33)**	**HAC (*n*=33)**	**HTLV-1 (*n*=66)**
−336	A	0.7031	0.8030	0.9091	0.8561
	G	0.2969	0.1970	0.0909	0.1439
	AA	0.5625	0.6061	0.8182	0.7121
	AG	0.2813	0.3939	0.1818	0.2879
	GG	0.1563	0.0000	0.0000	0.0000
−332	A	0.0000	0.0152	0.0455	0.0303
	G	1.0000	0.9848	0.9545	0.9697
	AA	0.0000	0.0000	0.0000	0.0000
	AG	0.0000	0.0303	0.0909	0.0606
	GG	1.0000	0.9697	0.9091	0.9394
−201	T	0.0156	0.0455	0.0303	0.0379
	G	0.9844	0.9545	0.9697	0.9621
	TT	0.0000	0.0000	0.0000	0.0000
	TG	0.0313	0.0909	0.0606	0.0758
	GG	0.9688	0.9091	0.9394	0.9242
−139	A	0.4063	0.2273	0.1818	0.2045
	G	0.5938	0.7727	0.8182	0.7955
	AA	0.2188	0.0606	0.0000	0.0303
	AG	0.3750	0.3333	0.3636	0.3485
	GG	0.4063	0.6061	0.6364	0.6212

**Table 5. t5:** *CD209* haplotype frequencies among HTLV-infected patients and controls

**Haplotype**	**Nucleotide at position**				**Haplotype frequency**			
	**−336**	**−332**	**−201**	**−139**	**HAM/TSP**	**HAC**	**CTRL**	**HTLV-1**
H1	A	G	G	G	0.5757	0.6969	0.2969	0.6363
H2	G	G	G	G	0.1515	0.0757	0.2812	0.1136
H3	A	G	G	A	0.2121	0.1515	0.4062	0.1818
H4	G	G	T	G	0.0454	0.0151	0.0156	0.0303
H5	A	A	G	A	0.0151	0.0303	0.0000	0.0227
H6	A	A	G	G	0.0000	0.0151	0.0000	0.0075
H7	A	G	T	G	0.0000	0.0151	0.0000	0.0075
